# General Hospitals and Infectious Diseases

**Published:** 1897-10-09

**Authors:** 


### GENERAL HOSPITALS AND INFECTIOUS DISEASES.

Certain difficulties which have arisen at Swansea have brought into prominence a matter of very considerable importance in hospital management, viz., the question of admitting cases of infectious disease to general hospitals. Time was when the hospital was looked upon as the proper place for almost every sort of ailment. In England small-pox has by general consent been excluded for a long time back, but until within a comparatively recent date all the other forms of fever were taken in. Even up to the present day fevers are admitted to most of the general hospitals in Dublin, several of which, in setting forth their advantages as places for medical study, make a special point of the fact that they have special wards for the reception of such cases.

### Irish Experience.

English notions receive rather a shock on finding that the returns, for example, from Sir Patrick Dun's Hospital classify the in-patients as follows: Fever, 165; maternity, 381; accident and other cases, 905. We have become so used to the idea that fever must be isolated that it is difficult to realise that in the capital of the sister isle they are at the present moment still discussing the question whether or not to erect isolation hospitals after the English model, and that high medical sanction is still given to existing arrangements, according to which fever cases are treated in the city hospitals. It would seem that in Dublin they use their hospitals for the treatment rather than for the isolation of fevers. Not only are the two fever hospitals, into which small-pox cases have also been received, situated in somewhat crowded parts of the city, but there are no fewer than seven fever departments in general hospitals. In a discussion on the subject which took place at the Royal Academy of Medicine in 1895, while it was generally admitted that isolation should be enforced as regards small-pox, the same view was by no means accepted in reference to the other infectious diseases. One speaker said that for the last thirty years he had been connected with the Adelaide Hospital, where they had always taken infectious diseases of all kinds except small-pox. This, by-the-bye, is a general medical and surgical hospital, containing children's wards, and giving special courses of instruction on diseases of women. During that time he had known only two patients who had contracted an infectious disease in the hospital. One was a patient with enteric fever, who caught typhus. The other was also suffering from enteric, and he caught scarlet fever. In each case the patient that gave the disease was lying in the next bed. There had never been a spreading of scarlet fever from the fever hospital to the main hospital, which was a few yards from it. Many other speakers followed in the same vein.

### Statistical Experience.

But if we leave mere opinions and inquire what is the total result of the treatment of fever in Dublin, including its treatment in general hospitals in connection with medical schools, we find that, notwithstanding an economic condition of population and a state of subsoil drainage, neither of which can conduce to health, the zymotic death-rate for the past six years (ending December 31st, 1896) was only 2·3 per 1,000, a rate which compares favourably with many English towns, the average in 1896 for the 33 large towns being 2·86, while London stood at 3·14 and Salford at 4·10.

In mentioning these facts we do not wish it to be understood that we approve of the Dublin system. We think, however, that they are sufficient to make it clear that we must enter upon the consideration of the question of the admission of infectious diseases to general hospitals with an open mind, and that we must not allow ourselves to be swept away in the current of the apparently simple and easy doctrine that, because a malady is infectious, it must be kept at arm's length.

### English Experience.

In Stevenson and Murphy's "Hygiene" Mr. Howse writes: "With due care as regards number, cases of typhus in former epidemics have been placed and treated successfully without spread of the malady in the large wards at Guy's amongst the other patients. In the case of typhoid and diphtheria a certain number of such cases are still admitted into the general medical wards with favourable results. As regards typhoid, the spread of the malady among surrounding patients is almost unknown, if the before-mentioned proportion of cases is duly maintained. It is very many years since an epidemic of typhoid has occurred in a Guy's ward, and when that took place it was due to accidental overcrowding of typhoid cases into one ward. As regards diphtheria, the risk of the spread of the disease is greater, but more amongst those nursing the case than amongst the ordinary patients, though even the latter is not unknown."

Again, in the evidence given before the Royal Commission on Metropolitan Hospitals in 1891 (Minutes of Evidence, March 19th, 1891), Dr. John Curnow, King's College, says: (Q. 18,996)—"We have at King's a standing rule of admitting ten cases" (of infectious disease). (Q. 18,997)—"Except small-pox, we take in anything that is bad." (Q. 18,997)—"I never knew infectious diseases spread in the hospital except in the one case of typhoid just now referred to by the matron, and that was not clear." (Q. 19,001)—"Are they (speaking of cases of scarlet fever) in the same wards as the ordinary patients?—Yes, unless there is anything special in the cases."

Before the same Commission Mr. Melhado, of the Middlesex Hospital, stated that a limited number of cases of diphtheria were admitted to general wards, no separate ward being provided. The disease had not spread in general wards, nor had it been contracted by nurses.

Mr. Michelli, of the Seamen's Hospital, also stated that many cases of diphtheria were admitted, and were not kept in separate wards unless the medical officer thought it absolutely necessary. It had not spread to either nurses or patients.

### Accidental Admissions of Infectious Cases.

We think, however, that no one would nowadays knowingly admit cases of measles or scarlet fever among other children. At the same time, no hospital can go on for long without accidentally admitting a child who is sickening for one or other of these diseases, and it sometimes becomes an anxious problem to know what to do. So far as concerns scarlet fever, the problem is greatly simplified in London by the fact that the Asylums Board will at once remove such cases to one or other of their admirable hospitals, where they will be under good professional supervision. But with measles, which is not removable to these hospitals, and also with scarlet fever cases in towns where the isolation hospitals are less well managed, the difficulty is great, so great, in fact, that we cannot admit that any hospital is fully equipped unless it possesses isolation wards in which such cases can be adequately dealt with. Where, as in the case of small cottage hospitals, such a permanent provision might seem in excess of what is required for an occasional accident, arrangement should certainly be made beforehand with some neighbouring householder, in whose house there are no childen, to receive such a case and its nurse without delay if the necessity should arise.

Diphtheria comes under another category, for in consequence of the urgent necessity for surgical intervention such cases must often require to be taken in during their most infectious stage. It becomes very necessary then to inquire how far this may be done without undue risk to the other patients.

### Post Scarlatinal Diphtheria.

The comparative frequency with which diphtheria follows scarlet fever (amounting in 1896 to 4 6 per cent. of the 15,176 cases admitted to the hospitals of the Metropolitan Asylums Board) suggests at once that under no circumstances should patients suffering from diphtheria be admitted to the general wards. But a more careful examination of the matter shows that the incidence of diphtheria in cases of scarlet fever must not be taken as representing the effect of the same intensity of infection on other people. It is a very striking thing to observe how the liability to post scarlatinal diphtheria increases week after week until the fifth, when it attains its maximum, and then gradually falls (Medical Supplement to Asylums Board Report for 1896), pointing, as this almost certainly does, to the fact that the liability to diphtherial infection after scarlet fever is something special to that disease, gradually increasing up to a certain point and then dying away.

### Diphtheria and Croup.

It is very difficult to express in a statistical form the liability of diphtheria to spread in an ordinary hospital ward where proper precautions are taken; but what is quite certain is that in a very large number of instances the admission of a single case in a ward has not been followed by any harm. We do not here refer to children's wards. The great majority of cases in which tracheotomy is required in children, although they may be spoken of as "croup," are really cases of diphtheria; yet it is the rarest thing for others in the ward to catch the disease. The danger seems to come in when there are several cases, and whenever that happens they certainly should have a ward to themselves. No doubt, if the nursing were not done with care the disease might spread, but just the same might be said of profusely suppurating wounds or deep and septic abscesses, all of which must needs be admitted occasionally, and for a time, at least, must be a source of risk; and if a hospital is sufficiently well managed to prevent the spread of suppuration, we may be pretty sure that an occasional diphtheria case need do no harm.

### Impossible to Select Cases.

It may be granted that if a general hospital could pick and choose its cases it could make itself a safer place for those who were lucky enough to get in, and on the other hand it could by the same means ensure reasonable safety by a less perfect type of nursing than is necessary at present.

But in practice it is impossible to refuse cases that are brought up, may be, from distant places. It is essential then that hospitals should be so conducted as to be able to admit almost every class of case that is likely to apply, and if a hospital is so organised that it can safely take in septic cases, a case or two of diphtheria will not matter. This is the point which really has to be recognised, that diphtheria is but one of a class of infections to the occasional incursion of which every general hospital is liable, and that no institution can properly or safely claim the title of a "general" hospital unless it is so arranged, both in regard to construction, nursing, and general management, as to be able to accept these various more or less infectious cases without any serious risk to its inmates.

### Typhoid Fever.

With how much more force then does the same argument apply to cases of typhoid fever. We do not say that the infection of typhoid never does spread to nurses or even to other patients. But what we do feel sure of is that when such an event occurs it is in consequence of some slip in the nursing, some error in the management, some little laxity which if it took place all round would make the hospital an unsafe place in regard to other infections besides that of typhoid fever. In these other infections, however, the evil might not be so obvious, while if typhoid is caught there is no doubt about it. The presence of a few cases of typhoid fever is then not only a good test of, but a great incentive to careful nursing. So long as the nursing is good and the hospital in a proper sanitary condition such cases do no harm, but they soon tell the tale if anything is wrong, and the first indication often is an infection of the nurses. From a purely patient's point of view this ought to be satisfactory. Passengers travel all the more happily for the knowledge that the man who drives the engine is in front.

### Tuberculosis.

The problem we are discussing is very much widened by the proof which has of late years been given as to the infectious nature of tuberculosis. No one can any longer doubt that the origin of tuberculous diseases, including ordinary phthisis, is an infection derived directly or indirectly from another patient. Consumption and other forms of tuberculosis must then be placed among the infectious diseases, and hospital managers have to face the problem, What is to be done? A little consideration, however, will show that what is to be done is not to refuse the cases, but to take care that their hospitals are so well managed that, infectious as these diseases may be, they shall not spread. Just as in typhoid fever and in diphtheria, so in phthisis the manner in which and the routes by which the infection might be disseminated are quite well known, and are capable of being brought well under control. We have also overwhelming proof that in well-conducted hospitals, even when filled with tuberculous patients, the disease does not spread, and there can hardly be a doubt that a consistent application of the same care to the more chronic infections as is now enforced in dealing with the acute ones will rob them of all their dangers. Hospital managers, then, may well be urged not to rush to the easy expedient of refusing patients merely because they are suffering from diseases which are included in the class "infectious," but to keep their standard of hospital work at such a level as will enable them safely to enlarge to the utmost the range of diseases which may be admitted without risk. Undoubtedly that range is already larger than it was some years ago.

